# Religious exception for vaccination or religious excuses for avoiding vaccination

**DOI:** 10.3325/cmj.2016.57.516

**Published:** 2016-10

**Authors:** Gordana Pelčić, Silvana Karačić, Galina L. Mikirtichan, Olga I. Kubar, Frank J. Leavitt, Michael Cheng-tek Tai, Naoki Morishita, Suzana Vuletić, Luka Tomašević

**Affiliations:** 1 Healh Care Center of Primorsko-Goranska County, Rijeka, Croatia; 2 Department of Social Sciences and Medical Humanities, University of Rijeka School of Medicine, Rijeka, Croatia; 3 Hotel Sveti Križ, Čiovo, Croatia; 4 Saint-Petersburg State Paediatric Medical University of Healthcare Ministry of the Russian Federation, Saint-Petersburg, Russian Federation; 5 Saint-Petersburg Pasteur Institute, Saint-Petersburg, Russian Federation; 6 Kushiro International Summer School and Conference in Bioethics, Kushiro, Hokkaido, Japan; 7 Chair professor, Medical Humanities/Bioethics, Chungshan Medical University, Taichung, Taiwan; 8 Hamamatsu University School of Medicine, Hamamatsu, Japan; 9 Department of Moral Theology, University of Osijek, Catholic Faculty of Theology, Osijek, Croatia; 10 Department of Moral Theology, University of Split, Catholic Faculty of Theology, Split Croatia

Vaccination is considered to be one of the greatest public health achievements in the 20th century, which has helped to build a society free of vaccine preventable diseases and save lives of millions children across the globe (1). However, in the 21st century, pediatric practice in the western world witnesses an era of vaccination refusal (2). Pediatricians, infectious disease experts, and public health professionals ask themselves why and how “the greatest achievement of public health” became a medical procedure that frightens parents across the globe. Many parents are seeking a legal way to avoid vaccinating their children. The legal systems of some countries predict legal vaccination exemptions. One of the most usual reasons for exemption are medical reasons, followed by the religious, social, and philosophical reasons (personal belief, conscience objection) (3-7).

2013 and 2015 were marked by an outbreak of vaccine-preventable diseases such as measles and pertussis (2,8-11). These events triggered a worldwide debate regarding vaccination and legal exemption of vaccination and its possible consequences such as social distancing, exclusion from school during a disease outbreak, absence from work, etc (12,13).

Religion influences decisions on vaccination (14-16), and religious objection is often used by parents as an excuse to avoid the vaccination of their children (5,17). Some studies show that the number religious exemptions has been increasing (18), leading to vaccine preventable disease outbreaks (10) such as mumps outbreak in a protestant orthodox group in The Netherlands. Shrivastwa et al (19) found religion as predictive factor of children’s vaccination status in India. Compared to Hindus, Muslim children had greater chance of being under-vaccinated or unvaccinated compared with the vaccinated children.

In this article we would like to explore whether different religious beliefs are, in itself, real exception for vaccination or they are just a parents’ excuse to avoid vaccination.

## The view of Catholicism

The most morally questionable issue regarding vaccination in Catholicism is using cell lines derived from a voluntary aborted fetus. The Moral Reflection On Vaccines published by the Pontifical Academy for Life (20) suggests that these vaccines should be avoided and proposes a search for alternatives. The examples of such vaccines are cell lines WI-38 (Winstar Institute 38) and MRC-5 (Medical Research council 5), several live vaccines against rubella (Meruvax, Rudivax, M-R-VAX), and vaccines against hepatitis (A-VAQTA and HAVRIX), chicken pox (Varivax), smallpox (AC AM 1000), and poliomyelitis (Polivax) (20,21). In the case where no alternative vaccine is available, the use of the existing vaccine is morally acceptable in order to avoid serious risks for children and for the whole population (especially pregnant women). The moral acceptability of using this vaccine should be comprehended as “passive material cooperation” and “active material cooperation” too, which is cooperation with immoral action without evil intention, permitted only in the case of “*extrema ratio*,” that is in the case of extreme situations such as saving the lives of children. The document also suggests to parents to oppose participation in such medical procedures by their appeal by “objection of conscience” or to seek alternative sources of effective vaccines. Besides this document, the Catholic Church's Magisterium discusses bioethical issues with respect to forbidden sources of human biological materials in two further documents. *Dignitas personae* (22), n. 34-35 speaks of the illicit origin of human sources of biological material, founding its opinions on the dignity of the person, emphasized in the documents *Donum vitae* (23) (I, 4) and *Evangelium Vitae* (24). In the case where ethically acceptable sources of vaccines are not available, it is necessary to weigh the vital importance and the risk of no vaccination. In these cases it becomes also allowed to use, even the, “morally inadvisable” vaccines (21).

The Catechism of the Catholic Church (25) does not cover the topic of vaccination directly. Indirectly, there are a few canons that could be applied to vaccination issue. The Church recognizes the ability of human intellect to meet the God (canon 39), which is the foundation for the dialog with other religions, philosophy, and science. The canons 1939-1943 emphasize the virtue of solidarity in the world. By spreading spiritual values, the Church has throughout the centuries helped to create better social and cultural conditions for living among different nations. Catholicism should emphasize the importance of taking the risk of side effects of vaccination to strengthen solidarity with other humans. By taking this risk, people participate in the protection of the entire society, including those who cannot be vaccinated because of medical contraindications or have been vaccinated but without adequate immunogenic response (20).

## Orthodox view

To clarify the Orthodox view on the vaccination process, we chose the example of Russia, a country where the orthodox religion is the leading religion and where historical perspective plays a special role in public, social, and cultural life. The Russian Orthodox Church absolutely acknowledges that vaccination is the main way to achieve progress. However, according to official statistics in Russia, annually 3%-5% of the population refuse to be vaccinated.

Refusal of vaccination and its causes cannot be clear without being familiar with the history of the anti-vaccination lobby. This history begins in 1988 with the article “Well, You Will Think, That It Is Only a Prick?” (26). This article claims that vaccination is not just a prick and that it causes serious complications. At present, the tribune of the anti-vaccination movement is the internet, which can provide access to the general population without demanding a true scientific assessment of efficiency and risks of vaccination. One of the motives used by opponents of vaccination is speculation in religious beliefs. Recently the anti-vaccination movement began to spread actively in monasteries, churches, and through a video production. An essentially medical question became “a bone of contention” among the believing people.

This all prompted the official Russian Church to issue an opinion about the topic of vaccination. In September 2008, the department of church charity and social service, the Synod organized a round table “Vaccine’s Prevention of Paediatric Problems and Ways of Making the Decision” (27,28). The Synod’s final document states: “Vaccination is a powerful tool of prevention of infectious diseases, some of which are extremely dangerous. In some cases inoculations really cause complications that are most often connected with violation of the rules of vaccination, such as its use on weakened children.” The Russian Orthodox Church condemned anti-vaccination promotion and forbade the distribution of anti-vaccination literature and audio-video material in its monasteries and temples (29).

The position of orthodox doctors and philosophers was reflected in the statement of Church Public Council on Biomedical Ethics of the Moscow Patriarchy and in the statement of the Department of Church Charity and social service of the Moscow Patriarchy and the Ministry of Health and Social Development of the Russian Federation (30). These documents unambiguously state that vaccination is a necessary modern measure of infectious diseases prevention, the refusal of which can lead to serious consequences.

At the same time it was noted that some aspects of vaccination demand additional attention. The Russian public has shown concern regarding vaccines against rubella, hepatitis A, and chicken pox, which are produced from the diploidic cells from aborted embryos. There are alternative (so-called “ethical”) rubella vaccines received from the cellular line of a rabbit (Japan), hepatitis A vaccines, made from the cellular culture of the monkey (Vero, Japan). However, application of these vaccines is only beginning and it is not widely available, so the diploidic vaccines against these diseases are mostly used.

## Protestant view

In Protestantism, there are various denominations without a supreme leading moral figure, such as the Pope in the Catholic Church. Protestantism accentuates individual freedom and gives parents the right to decide whether to vaccinate their children or not. According to Ruijs (16,17), Orthodox Protestant parents who refuse vaccination on religious grounds (17) claim that vaccination is an act of interfering with divine providence. Those who actually vaccinated their children consider the side-effects of vaccination as a God’s sign that they made a wrong decision. On the other hand, pro-vaccine parents believe vaccination to be a gift of God (17). Rujis also found that the religious leaders from the same denomination had different standpoints on vaccination: some do not address the topic of vaccination because it is generally accepted, the others deliberately leave the choice to the members of their congregation, and the third address the negative connotation regarding vaccination in their religious work (16).

## Jewish view

Israeli legislation has been traditionally influenced by religious law in the matters of birth, marriage, and death. However, Jewish theology tells us nothing explicit about today's medicine. Vaccination was unknown in Biblical and Talmudic times, but methods for preserving health and life, particularly cleanliness, were known. God made the man not only to His physical image and likeness, but to His mental image and likeness. When God commanded mankind to “be fruitful and multiply,” He left it up to people to decide how to do this. It is clear, however, that we cannot be fruitful and multiply unless we are healthy. We must use our minds, our power of thinking to decide how to preserve our health. These were the seeds of preventive medicine. We use the intelligence that God gave us to go beyond the raw nature which God created, and to preserve the health which we need to obey His commandments. Vaccination is one of the practices we developed from these seeds. It should be considered in the sense of *Pikuakh nefesh* – the act that saves lives (31), or the protection of the children and neighbors.

The distinguished religious Jewish organization, the Orthodox Union “strongly urges all parents to vaccinate their healthy children on the timetable recommended by their pediatricians” (32).

Although the Israeli law does not require vaccination, the government is trying to exercise pressure by denying child allowance payments to parents who do not vaccinate their children. This step has attracted opposition on the grounds of interference with individual rights. It is, however, not a matter of parents’ rights, but of the children’s right to health. The controversy has not yet been settled (33). When children are concerned, and serious risks to health or life are involved, it is irresponsible to ignore the almost universal weight of medical opinion. Children should be vaccinated, as almost all Israeli parents agree with it (34).

## Islamic view

The *Qur'ān* and the Islamic tradition forbid the use of certain food – *haram* (pig flesh). Other animals are licit – *halal* – depending on how they die (31). This problem is reflected in medicine regarding the use of gelatin in medical products. If gelatin is derived from *halal* animal it is permissible to use it. If someone finds him or herself in a situation where there is no *halal* alternative, the person is not guilty of using no-*halal* option based on the “law of necessity.” The vaccines are important for medical purpose, not for diet, therefore *haram* ingredients could be permitted (transformation of *haram* components to *halal* products). According to Islamic tradition, vaccination serves to protect life, to respect the principle of preventing harm (*izalat aldharar*), and public interest (*maslahat al ummah*). Vaccination protects others, which is why the law of necessity should be considered. It has a purpose in prevention, therefore its components cannot be judged as a diet (31).

## Buddhist view

Buddhism claims that life is one, which means that all forms of life are essentially related to one another and share a common essence. Even though there are different expressions of life, their lives are basically the same and they only differ in their external forms of being. Buddhism also believes in the Wheel of Rebirth, meaning that all forms of life will be reincarnated according to the karma they accumulated while living. Someday, in the process of reincarnation when all karma has been completely exhausted, the wheel of rebirth can be stopped. In order to reach this *Nirvana*, every Buddhist must carefully observe the 8-fold Path and the Ten Precepts that help prevent any accumulation of karma. These precepts include: not taking life, not stealing, being chaste, not lying, not drinking intoxicants, etc (35). The first of the five precepts in Pali reads as *Panatipata veramani sikkhapadam samadiyami* meaning that “I undertake the training rule to abstain from taking life.” Here the word *pana* refers to any living being that has breath and consciousness.

The Mahayana Brahajala Sutra explains the first precept in this way: “A disciple of the Buddha shall not himself kill, encourage others to kill, kill by expedient means, praise killing, rejoice at witnessing killing, or kill through incantation or deviant *mantras.* He must not create the causes, conditions, methods, or karma of killing, and shall not intentionally kill any living creature” (36,37).

Modern Buddhists will generally use vaccines to make sure their health is protected. But according to the essential teaching of Buddhism, if the vaccine is derived from any life form its use is debatable. The first of the Ten Buddhist Precepts is “not taking life.” However, early Buddhism was never confronted with the question whether a fetus is a life form. Buddhism basically forbids any act that will lead to the destruction of any potential life. Therefore, Buddhism requires its followers to treat all life kindly (38).

On the other hand, Buddhist biomedical researchers who experiment on life forms believe that the purpose of biomedical research is to save rather than to sacrifice life. Buddhist biomedical researchers do the experiments for the love of life, for instance, they experiment on the donated tissues or samples, thereby accumulating no bad karma. The modern view of Buddhism will stress the importance of saving life rather than taking life (39). Generally speaking, Buddhist teaching is rather conservative in terms of using any life form to create vaccine.

## Japanese view

There have been many religious forms in Japan. However, Japanese people do not have a clear belief system called “religion.” So that “Japanese religion” means “Japanese metaphysical common sense.” This metaphysical sense has been formed by integrating and mixing Buddhism, Taoism, and Confucianism, based on the indigenous Shinto (40-42). Here “metaphysical” means “a way of seeing or thinking of the universe or the total reality,” which is the core of religion. Religious forms are various, but their metaphysical core is consistent.

The universe for Japanese people is a moving network of various relations of things and actions, like an infinite living system. They believe in an unknowable and willful entity reflecting the universe like a virtual focus in a mirror, which has been called *Kami* (gods), *Hotoke* (Buddha), or *Ten* (Heaven). This mysterious entity orders and gives people the whole necessary connections of universe, which is called *Michi* (Tao or Way) or *Ri* (Logos or Ratio). Based on their religious common sense background, Japanese people accept all relations of things and actions as they are and feel very familiar with everything relating to them. Moreover, with gratitude, they hold memorial services for used tools or for sacrificed laboratory animals (43). Therefore, they tend to reject all biomedical practices, technologies, or effects considered as unnatural. This is very obvious in the case of organ-transplant and vaccination. This “unnatural” implies the complex feelings of certain deviation or excess from the standard course of things.

Since Meiji Revolution (1868), Japanese people have basically acknowledged modern biomedicine, and they have gradually accepted vaccination as its symbol. In 1948, new Japanese government made vaccination mandatory. Thus, after 1962, vaccination has been practiced collectively and compulsory. But, in 1994, it was suddenly proclaimed optional under the pressure of the public taking side effects as dangerous.

## Concluding remarks

Vaccination refusal among the parents of pediatric population is emerging globally, regardless of religious or political background or geographical location. In many countries legal systems advise how to react to vaccination refusal (44). For example, in Croatia vaccination is mandatory, the law is clear, but the practice of vaccination and the court judgments are not standardized. The legislators are unlikely to enact legal limitations of religious or philosophical exemption (4,5).

The number of vaccination refusals based on religious exemption is increasing. The question is whether religious freedom is a threat to public health, in this case to the vaccination system (45).

There are many publications regarding the religious exception of vaccination (6,14,15) based on the rights of religious freedom. Most of these publications refer to religious exemption for immunization. However, religion can provide perspectives on vaccination that are rarely used in debates on this topic. For example, the notions of solidarity, risk sharing, or taking the risk of vaccination for those who cannot be vaccinated because of medical contraindication or because of their conditions.

Although in this paper the authors did not cover all religions, they reflect on religions and social environment of the society which they come from. The majority of religions respect life as a basic value and therefore oppose the use of vaccines derived from aborted human fetuses (Catholicism) or any form of life (Buddhism). But if these vaccines serve to protect many more lives they are permitted. Regarding this, we should not consider vaccination opposed to the theological base and values. Following this idea, religion is not in contradiction with vaccination and public health. It is only individual parents or religious leaders and their questionable interpretation of religious practices that are opposed to vaccination, no religion as such. In order to protect vaccination from the questionable religious interpretation we should bring closer to the public the basic theological perspective. The society of the 21st century, just as many societies and cultures in the history of human civilization, use religion as an excuse for wars, discrimination, and now for vaccination refusal. The question is whether the public is aware of the teachings of their religion on these issues. One of the first steps in resolving the situation should be the appropriate communication (46-48) to illuminate the essence of theological perspectives regarding vaccination.
